# Arabinogalactan Utilization by *Bifidobacterium longum* subsp. *longum* NCC 2705 and *Bacteroides caccae* ATCC 43185 in Monoculture and Coculture

**DOI:** 10.3390/microorganisms8111703

**Published:** 2020-10-31

**Authors:** Yan Wang, Gisèle LaPointe

**Affiliations:** Department of Food Science, Canadian Research Institute for Food Safety, University of Guelph, Guelph, ON N1G 2W1, Canada; ywang62@uoguelph.ca

**Keywords:** arabinogalactan, *Bifidobacterium longum*, *Bacteroides caccae*, β-galactanase, α-L-arabinofuranosidase, β-L-arabinopyranosidase

## Abstract

Arabinogalactan (AG) has been studied as a potential prebiotic in view of stimulating bifidobacteria presence in the gut microbiota. However, bifidobacteria prefer fermentation of oligosaccharides to that of polysaccharides. The contribution of other gut bacteria may allow better growth of bifidobacteria on AG. β-galactanases and β-galactosidases are the main enzymes for the degradation of AG. Additional enzymes such as α-L-arabinofuranosidase and β-L-arabinopyranosidase are required to remove the arabinose side chains. All of these predicted functions are encoded by the genomes of both *Bifidobacterium longum* subsp. *longum* NCC 2705 and *Bacteroides caccae* ATCC 43185. However, neither strain was able to grow significantly on AG, with 25% (*B. longum* subsp. *longum* NCC 2705) and 39% (*Bac. caccae* ATCC 43185) of AG degraded after 48-h fermentation, respectively. In this study, the β-galactanase, β-galactosidase, α-L-arabinofuranosidase, and β-L-arabinopyranosidase from both strains were investigated. The extracellular β-galactosidases of both *B. longum* subsp. *longum* NCC 2705 and *Bac. caccae* ATCC 43185 were able to cleave the β-1,3; 1,4 and 1,6 linkages. However, the β-galactosidase activity of *B. longum* subsp. *longum* NCC 2705 was weaker for the β-1,4 linkage, compared with the β-1,3 and 1,6 linkages. The arabinose side chains of AG inhibited the cleavage of β-1,3 and 1,6 linkages by the endo-β-galactanase from both strains, and partially inhibited the cleavage of β-1,4 linkages by the endo-β-1,4 galactanase from *Bac. caccae* ATCC 43185. The α-L-arabinofuranosidase and β-L-arabinopyranosidase from both strains were unable to cleave arabinose from AG under the conditions used. These results show limited breakdown of AG by these two strains in monoculture. When cocultured with *Bac. caccae* ATCC 43185, *B. longum* subsp. *longum* NCC 2705 grew significantly better than in monoculture on AG after 6 h of fermentation (*p* < 0.05). The coculture showed 48% AG degradation after 48 h of fermentation, along with reduced pH. Furthermore, compared to monoculture of *Bac. caccae* ATCC 43185, the concentration of succinate significantly increased from 0.01 ± 0.01 to 4.41 ± 0.61 mM, whereas propionate significantly decreased from 13.07 ± 0.37 to 9.75 ± 2.01 mM in the coculture (*p* < 0.05). These results suggest that the growth and metabolic activities of *Bac. caccae* ATCC 43185 were restrained in the coculture, as the pH decreased due to the metabolism of *B. longum* subsp. *longum* NCC 2705.

## 1. Introduction

Colon microbes ferment dietary fiber to produce short chain fatty acids (SCFAs) that have many roles in promoting gut health [1]. The ratio of SCFAs produced varies with the metabolism of each gut bacterial species and carbohydrate state [2]. The SCFAs may reduce pH in the colon and results in shifting of the composition of gut microbiota, which is determined by the acid tolerance of gut bacterial species [2]. Many species of Firmicutes, which are Gram-positive and characterized with a low percentage of G + C, and *Actinomycetes* (high % G + C) show relatively greater acid tolerance, whereas the *Bacteroides* spp. have less acid tolerance [3].

Arabinogalactan (AG), which is a constituent of pectin, has been studied both in vivo and in vitro as a potential prebiotic in view of stimulating bifidobacteria in the gut microbiota [4,5,6]. However, the enzyme functions for AG degradation in gut bacteria, even though predicted from genome analysis, are still poorly understood from a functional viewpoint. There are two structural types of AG, type I and type II [7]. Type I AG consists of a β-1,3 and 1,4 linked galactan backbone, whereas type II AG has a more complex structure consisting of a β-1,3 linked galactan backbone with β-1,6 linked galactan side chains [8,9]. In addition, the backbone of type I AG and side chains of type II AG are substituted with α-arabinofuranose and/or, less frequently, β-arabinopyranose at the non-reducing terminus of side chains of both types of AG [7,8]. Gum arabic (GA) has a similar structure to type II AG, which includes a β-1,3 linked galactan backbone and β-1,6 linked galactan side chains with some arabinose substitutions [10].

AG can be utilized by many *Bacteroides* species, such as *Bacteroides thetaiotaomicron*, *Bac. uniformis*, *Bac. cellulosilyticus*, *Bac. ovatus*, and *Bac. caccae*, while *Bac. distasonis*, *Bac. eggerthii* and *Bac. fragilis* cannot [11,12]. *Bifidobacterium* species prefer fermentation of oligomers of relatively short degree of polymerization (DP), such as fructooligosaccharides (FOS) and galactooligosaccharides (GOS) [13]. Furthermore, bifidobacteria also have the ability to degrade arabinoxylan oligosaccharides [(A)XOS], but this activity is strain-dependent [14]. Many in vitro studies reported that some gut bacteria cannot degrade specific polysaccharides by themselves but take advantage of the metabolic products of other gut bacteria [15]. These products may be carbohydrate fragments and/or fermentation products such as SCFAs [16].

β-galactanases are the main enzymes for degradation of the backbone of AG, resulting in the release of galactose and/or GOS. Two kinds of galactanases, exo-β-1,3-galactanase and endo-β-1,4-galactanase, have been cloned from *Bifidobacterium longum* and expressed in *Escherichia coli* [8,17]. Both enzymes are extracellular but the degradation of the AG backbone by both enzymes is inhibited by arabinosyl side chains [8,17]. However, the study did not demonstrate which linkage type was inhibited. Furthermore, endo- and exo-β-1,3-galactanases from *Bac. thetaiotaomicron* species have been characterized [10,18]. The exo-β-1,3-galactanase was also inhibited by arabinosyl side chains, but there is no information on whether endo-β-1,3-galactanases are inhibited in the same manner [10].

Given that arabinose side chains of AG may inhibit cleavage of the backbone of AG by some β-galactanases, extracellular arabinosidases may have an important role in removing arabinosyl side chains for efficient AG degradation. Two of the extracellular α–arabinofuranosidase genes were induced in *B. longum* subsp. *longum* NCC 2705 when cocultured on (A)XOS with *Eubacteria rectale* ATCC 33656 [19]. However, these results represent gene expression, not enzyme activity. The β-L-arabinopyranosidases have been cloned from *B. longum* and *Bac. thetaiotaomicron* and characterized by heterologous expression in *E. coli*, respectively [10,20]. The carbohydrate-active enzyme (CAZymes) database shows that the genome of most *Bacteroides* spp. encode α-arabinosidase genes, and the enzyme activities were determined for *Bac. thetaiotaomicron*, *Bac. plebeius*, *Bac. coprocola, Bac. barnesiae*, *Bac. salanitronis*, *Bac. gallinarum* and *Bac. intestinalis* [10,21,22,23]. However, the cellular location and substrate specificity of the enzymes have not been demonstrated.

The GOS released from AG can be transported into the bacteria or degraded to galactose outside the cell. A three-gene cluster encoding an ATP-binding cassette (ABC)-type uptake system for GOS has been shown in *B. breve* UCC 2003, which includes one GOS-binding protein and two permease proteins [24]. In addition, a total of nine β-galactosidase genes from *B. bifidum*, *B. infantis* and *B. breve* have been isolated and characterized [24,25,26]. Two of them are predicted to be extracellular enzymes, and seven of them are intracellular enzymes. Furthermore, β-galactosidase activities have also been reported from *Bac. polypragmatus*, *Bac. fragilis*, *Bac. thetaiotaomicron* among *Bacteroides* species [27,28,29].

Compared to oligosaccharides, AG has longer DP and more complex monomer saccharide units, which may exceed the substrate specificity of bifidobacterial enzymes [30]. One study reported that nine *B. longum* strains were incapable of significant growth on AG in pure culture [24]. In general, *B. longum*, *B. breve*, and *B. bifidum* are dominant in infants, while *B. catenulatum*, *B. adolescentis* and *B. longum* are isolated from adult fecal samples [31,32]. In 42 fecal samples from healthy Belgian adults, among bifidobacterial species, *B. longum, B. adolescentis*, and *B. catenulatum* were present in 90%, 79% and 38% of samples, respectively [33]. Therefore, the observed bifidogenic effect of AG in vivo and in vitro (fecal inoculation) studies is likely due to the cross-feeding of products released by other AG-degrading bacteria, such as *Bacteroides* species.

The aim of this study was to investigate the specificity of these enzymes from gut bacteria strains in order to understand their contribution to AG degradation. Furthermore, a better understanding of the metabolic interaction between bifidobacteria and *Bacteroides* spp. in the presence of AG is required. Both *B. longum* subsp. *longum* NCC 2705 and *Bac. caccae* ATCC 43185 strains were isolated from human feces, and the whole genome sequences of both strains have been published [34]. However, the enzyme activities for AG degradation from both strains have not been characterized. In this study, the characteristics of AG degradation enzymes from *B. longum* subsp. *longum* NCC 2705 and *Bac. caccae* ATCC 43185 were determined, which included quantification of activity, cellular location, and substrate specificity. The possible metabolic cooperation between *B. longum* subsp. *longum* NCC 2705 and *Bac. caccae* ATCC 43185 during AG fermentation was investigated.

## 2. Materials and Methods

### 2.1. Carbohydrate Substrates

Larch wood AG (average molecular weight (MW): around 20 kDa (after autoclaving)), GA from acacia tree and *p*-nitrophenyl (*p*NP) substrates were purchased from Sigma-Aldrich (Oakville, Canada). Potato galactan, de-arabinosylated potato galactan, and other small MW substrates (β-1,3-galactobiose, β-1,4-galactobiose, β-1,6-galactobiose, α-1,5-arabinobiose, α-1,5-arabinotriose, α-1,5-arabinotetraose, 2^3^-α-L-arabinofuranosyl-xylotriose (A2XX) and 3^2^-α-L-arabinofuranosyl-xylobiose (A3X)) were obtained from Megazyme (Burlington, Canada).

De-arabinosylated AG (de-AG) and de-arabinosylated GA (de-GA) were prepared by mild acid degradation, as described previously with minor modifications [35]. Briefly, AG (90 g) or GA (90 g) were dissolved in water to a final volume of 450 mL, respectively. After heating to 95–98 °C with continuous stirring, 50 mL of trifluoroacetic acid (2.5 M) (Sigma-Aldrich, Oakville, Canada) were added to the solutions and stirred continuously at 90–95 °C for another 2 h. After incubation, the solutions were rapidly cooled on ice for 5 min, and the pH was adjusted to 7.0 using 5 M NaOH. The supernatant was collected by centrifugation at 15,000× *g* for 5 min at room temperature, followed by filtration through a binder-free glass microfiber filter (GF/A grade; Whatman, Maidstone, UK). The de-AG and de-GA were precipitated with 2 volumes of absolute ethanol overnight at 4 °C and washed twice with absolute ethanol. The washed de-AG and de-GA precipitates were dried in the vacuum oven at 50 °C overnight. To remove fragments of sugar units, the de-AG, de-GA and de-arabinosylated potato galactan were dissolved in water and dialyzed in 3500 Da cut-off dialysis cassettes (Thermo Scientific, Mississauga, Canada) against water for 3 days at 4 °C, followed by drying in the vacuum oven at 50 °C overnight.

### 2.2. Media, Strains and Growth Conditions

The MRS medium (Oxoid, Nepean, Canada) was supplemented with 0.05% (*w*/*v*) L-cysteine·HCl for growth of *B. longum* subsp. *longum* NCC 2705. The yeast extract-casitone medium supplemented with short chain fatty acid (YCFA) contained (per liter): 10 g casitone, 2.5 g yeast extract, 4 g NaHCO_3_, 1 g L-cysteine·HCl, 0.45 g K_2_HPO_4_, 0.45 g KH_2_PO_4_, 0.9 g NaCl, 0.9 g (NH_4_)_2_SO_4_, 0.09 g MgSO_4_·7H_2_O, 0.09 g CaCl_2_, 0.001 g resazurin, 0.01 g hemin, 0.00001 g biotin, 0.00001 g cobalamin, 0.00003 g *p*-aminobenzoic acid, 0.00005 g folic acid, and 0.00015 g pyridoxamine. The following SCFAs were added (final concentrations): acetate (33 mM); propionate (9 mM); isobutyrate, isovalerate, and valerate (1 mM each). The SCFAs were excluded when appropriate, which was referred to as YC medium. The carbon sources were added at a final concentration of 0.5% (*w*/*v*) to YCFA or YC medium. The final pH of the medium was adjusted to 6.3 ± 0.1 by using 1 M NaOH before sterilization at 121 °C for 15 min. Sterilized medium was cooled and filter-sterilized solutions of thiamine and riboflavin were added to give a final concentration of 0.05 μg/mL of each.

*B. longum* subsp. *longum* NCC 2705 was obtained from the Nestlé Research Centre (Lausanne, Switzerland); *Bac. caccae* ATCC 43185 was purchased from the American Type Culture Collection (Manassas, VA, USA). *B. longum* subsp. *longum* NCC 2705 was stored in MRS-cysteine while *Bac. caccae* ATCC 43185 was stored in YCFA-glucose, both at −80 °C with 24% (*v*/*v*) glycerol. *B. longum* subsp. *longum* NCC 2705 and *Bac. caccae* ATCC 43185 were resuscitated from frozen stocks in MRS-cysteine and YCFA-glucose, respectively. Subsequently, the cultures were inoculated at 3% (*v*/*v*) into YC medium supplemented with the carbon source and YC medium without carbon source as a control. For coculture, 3% (*v*/*v*) of each strain culture was inoculated in YC with the carbon source. All cultures were incubated anaerobically in an anaerobic chamber (Model UM-041, Ruskinn Technology, Sanford, USA) containing an atmosphere of 80% N_2_, 10% H_2_, and 10% CO_2_ at 37 °C. At selected fermentation times, optical density (OD) at 600 nm and pH of harvested culture broth were measured by using a spectrophotometer (Model DU530, Beckman, Brea, USA) and a pH meter (Fisher Scientific, Nepean, Canada), respectively. All growth curves were repeated three independent times.

### 2.3. Analysis of Carbohydrate Degradation

The MW profile of carbohydrates in the spent culture media were analyzed by high-performance size exclusion chromatography (HPSEC). After 48-h fermentation, 1 mL of cell-free supernatants obtained through centrifugation at 14,000× *g* for 15 min were deproteinated with Carrez clarification reagent kit (Sigma-Aldrich, Oakville, Canada) following the manufacturer’s protocol. The protein-free supernatant or dextran standards (Sigma-Aldrich, Oakville, Canada) were mixed with the same volume of mobile phase (0.1 M NaNO_3_ and 0.01 M NaH_2_PO_4_; pH: 7.7), followed by centrifugation at 14,000× *g* for 15 min. The supernatant of the mixture was filtered through a 0.45-μm filter (Fisher Scientific, Nepean, Canada). Forty microliters of sample or standard were analyzed on a Shimadzu LC-10A HPSEC system (Shimadzu Corporation, Kyoto, Japan) equipped with a PL Aquagel-OH-Mixed M column (7.5 × 300 mm, 8 µm, Agilent Technologies Canada Inc., Mississauga, Canada) and refractive index (RI) detector. The temperature of the column and detector were set at 40 °C, and mobile phase at a flow rate of 0.3 mL/min. All instrument control, analysis, and data processing were performed using the Labsolutions platform (Shimadzu corporation. Version 5.82). Known MW (1 kDa, 5 kDa, 12 kDa, 25 kDa, 50 kDa and 80 kDa) dextran standards were used for mass approximation of AG present in the culture. The percentage of degradation was quantified by peak area at retention time between 27.8 and 32.2 min (50 kDa-5 kDa).

(1) Percentage of degradation=(1−48 h peak area/0 h peak area)×100%

### 2.4. Analysis of Enzyme Activity and Substrate Specificity

A volume of 1 mL of YC-AG broth culture was harvested after 16 h of fermentation and centrifuged at 14,000× *g* for 5 min at 4 °C. The cell pellets were washed twice with 500 μL of 0.2 M phosphate buffer (PB) (pH 6.3), disrupted by Bead Mill 24 (Fisher Scientific, Nepean, Canada) in 500 μL of the same buffer with 0.6 g of glass beads (0.1 mm, BioSpec Products, Inc., Bartlesville, USA) for 10 min, followed by centrifugation at 14,000× *g* for 5 min at 4 °C. The supernatant containing crude cytoplasmic enzymes was collected. The precipitate containing crude cell wall-associated enzymes was suspended in 500 μL of the same buffer after washing twice.

To quantify cell wall-associated and cytoplasmic enzymes, the *p*NP test was performed as described previously [36]. Briefly, 10 μL of the crude enzyme was mixed with 10 μL of 5 mM *p*NP substrates and 80 μL of PB buffer (0.2 M; pH 6.3), followed by incubation at 37 °C for 30 min. The reaction was stopped by the addition of 100 μL of 1 M Na_2_CO_3_, and liberated *p*NP was measured at 405 nm using a spectrophotometer (Multiskan^TM^ GO Microplate Spectrophotometer, Thermo Scientific, Mississauga, Canada). One unit (U) of enzyme activity was defined as the amount of enzyme that released 1 μmol of *p*NP per min at 37 °C. The enzymatic assays were conducted in triplicate on each extract.

The enzymatic substrate specificity was analyzed by thin-layer chromatography (TLC) as described previously [20]. Eighty μL of the crude enzyme was incubated with 20 μL of substrate (0.2 mg/mL in water) at 37 °C for 16 h. Ten microliters of the reaction products and standards were loaded on a TLC silica gel 60 aluminum sheet (Merck, Darmstadt, Germany) using a 7:1:2 (*v*/*v*/*v*) *n*-propanol-ethanol-water as mobile phase. The carbohydrate was visualized by spraying the plate with 180 mg orcinol in H_2_SO_4_ solution (water (5 mL): ethanol (75 mL): H_2_SO_4_ (10 mL)) and heating.

### 2.5. PMA Treatment and Droplet Digital PCR (ddPCR) Analysis

To enumerate viable cells, *B. longum* subsp. *longum* NCC 2705 and *Bac. caccae* ATCC 43185 in mono and co-cultures were treated with propidium monoazide (PMA) (Biotium, Fremont, USA) using a previously described method with minor modifications [19]. Briefly, 1-mL aliquots of culture were centrifuged at 14,000× *g* for 15 min at 4 °C. After removing the supernatant, 500 μL of sterile peptone water (1 g/L peptone, 0.5 g/L cysteine-HCL, pH 6.8) was added. The cell suspension was treated with 10 μL PMA (2.5 mM) and shaken in the dark for 5 min at room temperature. The PMA-treated cells were exposed in a UV light (PhAST Blue, GenIUL, Barcelona, Spain) for 15 min, followed by centrifugation at 14,000× *g* for 15 min at 4 °C. The PMA-treated cell pellets were washed by 800 μL of sterile physiological saline (0.85% NaCl), followed by centrifugation at 14,000× *g* for 15 min at 4 °C. The cell pellets were stored at −20 °C until DNA extraction. The DNA of both strains was extracted by using the DNeasy UltraClean Microbial Kit (Qiagen, Mississauga, Canada) following the manufacturer’s protocol. The DNA extracts were quantified and qualified using Qubit 4 (Invitrogen Canada Inc., Burlington, Canada) and stored at −20 °C until use.

The number of viable *B. longum* subsp. *longum* NCC 2705 and *Bac. caccae* ATCC 43185 in mono and co-culture were quantified by the ddPCR system following the manufacturer’s instructions. Briefly, a total of 25 μL of each reaction mixture contained 12.5 μL of QX200 ddPCR EvaGreen supermix, 5 μL of DNA, and 0.7 μL each of 10 μM forward and reverse primers (Table 1). Droplets were generated using QX200 Droplet Generator with a disposable cartridge. The cartridge was filled by 20 μL of each reaction mixture in the sample well and 70 μL of droplet generator oil in the oil well. After droplet generation, 40 μL of droplet suspension were transferred to a 96-well ddPCR plate and the plate was sealed with foil in a PX1 PCR Plate sealer at 180 °C for 5 s. The sealed plate was inserted into a C1000 Touch Thermal Cycler with the following amplification program: 95.0 °C for 10 min, 40 cycles at 95.0 °C for 30 s, the appropriate annealing temperature and time for each primer set (Table 1), and one cycle of 98 °C for 10 min with a 4 °C hold. The fluorescence was read using the QX200 Droplet Plate Reader, and data were analyzed using Quantasoft software. All of the equipment, materials and regents for ddPCR analysis were purchased from Bio-Rad (Mississauga, ON, Canada).

### 2.6. Quantification of SCFAs and Other Metabolites

The concentration of acetate, butyrate, lactate, formate, ethanol, propionate and succinate were determined through single-dimension proton nuclear magnetic resonance (1D 1H NMR) following a previously described protocol [38]. Briefly, culture samples were prepared first by serial filtration (1 μm, 0.8 μm, 0.45 μm, and 0.22 μm) (Whatman GE, Maidstone, UK). Filtered samples (630 μL) were mixed with 70 μL of internal standard, IS-2 Chenomx internal standard-DSS-d6 solution (Chenomx Inc., Edmonton, Canada), on the day of the analysis. Sample mixtures were transferred to 5-mm glass NMR tubes (NE-UL5-7, New Enterprises Inc., Vineland, NJ, USA) and analyzed by a Bruker Avance 600.13 MHz spectrometer with a triple resonance probe (TXI 600). The TXI 600 for scanning was performed in a single batch using the first increment of a 1D NOESY pulse sequence that had t_mix_ of 100 ms, 5.25-s acquisition time, 1-s relaxation delay, and a spectral width of 14 ppm. The spectra were analyzed by Chenomx NMR Suite 7.0-7.7 (Chenomx Inc., Edmonton, Canada). The compounds were determined through comparison with the compound library. A known concentration of internal standard was used for the quantification of compounds.

### 2.7. Analysis of Nucleotide Sequences

The enzyme coding genes were analyzed through the Integrated Microbial Genomes and Microbiomes web server (https://img.jgi.doe.gov). The IMG genome ID of *B. longum* subsp. *longum* NCC 2705 and *Bac. caccae* ATCC43185 are 637000031 and 640963023, respectively. Cellular localization of putative AG degradation genes was defined based on the PSORTb v3.0 web server (https://www.psort.org/psortb/).

### 2.8. Statistical Analysis

Statistical analyses were performed using Graph-Pad Prism 8. Means and standard deviations of three replicates were analyzed by Student’s two-tailed *t*-test (Figure 4 and Figure S1; Table 2) or Dunnett’s multiple comparisons test (Figure 5); where *p* < 0.05 was considered as significant.

## 3. Results

### 3.1. Analysis of Genes Coding for Potential AG-Degrading Enzymes

Three genes coding for AG degradation enzymes (β-galactanase, β-galactosidase and α-arabinosidase) were identified in the genome of both strains (Table S1). The *B. longum* subsp. *longum* NCC 2705 genome encodes one cell wall-associated β-galactanase gene that comprises a signal peptide and an LPXTG cell wall anchor domain. The genome of *Bac. caccae* ATCC 43185 also encodes one β-galactanase gene that comprises a signal peptide, but the PSORTb web server was unable to predict the enzyme cellular location, and a cell wall anchor sequence was not found. The *B. longum* subsp. *longum* NCC 2705 genome encodes four β-galactosidase genes, two of which are predicted to be cytoplasmic enzymes. A total of sixteen β-galactosidase genes are encoded in the genome of *Bac. caccae* ATCC 43185. Among these β-galactosidase genes, six are predicted to be cytoplasmic enzymes, whereas six β-galactosidase genes each encode a signal peptide, but the PSORTb web server was unable to predict the cellular location. The *B. longum* subsp. *longum* NCC 2705 genome codes for five α-arabinosidase genes, three of which are predicted to be cytoplasmic enzymes, whereas the *Bac. caccae* ATCC 43185 genome encodes only one α-arabinosidase gene.

### 3.2. Growth in Pure Culture on Seven Carbon Sources

To avoid the effect of SCFAs on cell growth, YC medium was used. Both strains can utilize major constituent sugar units of the AG, arabinose and galactose, for growth (Figure S1a,b). After 24 h of fermentation, the OD_600_ reached 4.07 ± 0.44 and 3.63 ± 0.38 when *Bac. caccae* ATCC 43185 grew on galactose and glucose, respectively. Both strains had less growth on AG compared to growth on each monosaccharide at the same concentration. In medium with AG, *Bac. caccae* ATCC 43185 reached an OD_600_ of 1.75 ± 0.12 after 24 h of fermentation, which was almost 2.5 times less than its growth on galactose (Figure S1b,d). *B. longum* subsp. *longum* NCC 2705 showed the highest growth on glucose (OD_600_: 1.28 ± 0.23), whereas the OD_600_ just reached 0.28 ± 0.08 when growing on AG after 24 h of fermentation (Figure S1a,c). Both strains achieved higher optical density on AG than on GA (Figure S1c,d). However, compared to GA (OD_600_: 0.60 ± 0.16), the de-GA (OD_600_: 1.16 ± 0.14) significantly stimulated the growth of *Bac. caccae* ATCC 43185 after 24 h of fermentation (*p* < 0.05) (Figure S1d).

### 3.3. AG Degradation Profiles after Fermentation

The main peak of autoclaved AG occurred between 50 and 5 kDa (Figure S2). The AG (50–5 kDa) can be degraded by both strains, as shown by the decreasing area of the 20-kDa peak and increased presence of peaks with longer retention time, indicating smaller degradation products (Figure S3). After 48 h of fermentation, 25% and 39% of AG (50 kDa-5 kDa) was degraded by *B. longum* subsp. *longum* NCC 2705 and *Bac. caccae* ATCC 43185, respectively.

### 3.4. AG-Degrading Enzyme Activities

Assays with *p*NP substrate showed that *B. longum* subsp. *longum* NCC 2705 and *Bac. caccae* ATCC 43185 had cell wall-associated α-arabinofuranosidase and β-arabinopyranosidase activity when they were cultured in the presence of AG (Table 2). Both cytoplasmic (0.1 ± 0.006 U/mg protein) and cell wall-associated (0.19 ± 0.027 U/mg protein) α-arabinofuranosidase specific activities of *B. longum* subsp. *longum* NCC 2705 were significantly higher than those of *Bac. caccae* ATCC 43185 (0.01 ± 0.001 U/mg protein and 0.09 ± 0.006, respectively) (*p* < 0.05). Both strains had cell wall-associated β-galactosidase activity, which was significantly lower than that of cytoplasmic β-galactosidase (*p* < 0.05). *B. longum* subsp. *longum* NCC 2705 had around 10 times higher cytoplasmic β-galactosidase activity (2.64 ± 0.159 U/mg protein) than that of cell wall-associated enzyme activity (0.22 ± 0.031 U/mg protein). Furthermore, the activities of both cell wall-associated and cytoplasmic β-galactosidase from *B. longum* subsp. *longum* NCC 2705 were significantly higher than those of *Bac. caccae* ATCC 43185 (*p* < 0.05).

**Figure 1:**
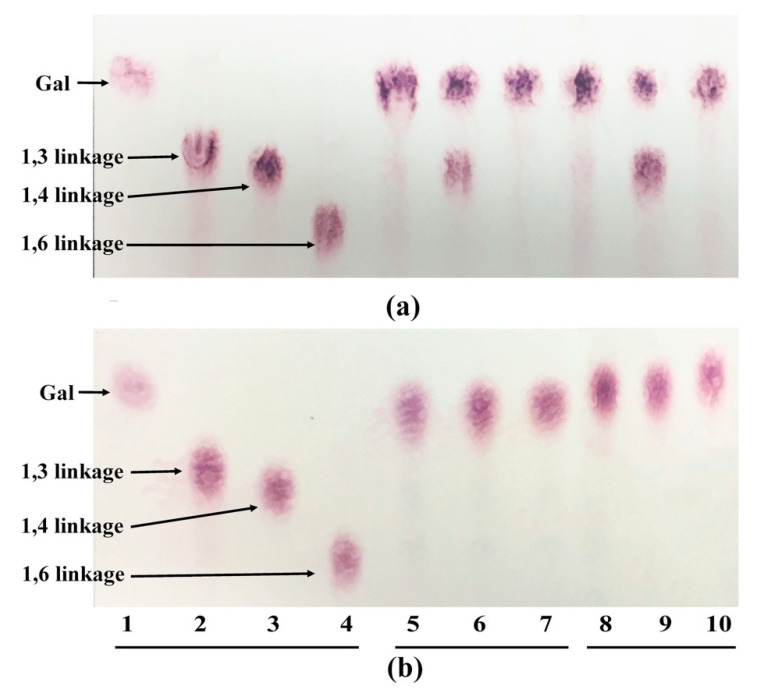
Thin-layer chromatography (TLC) analysis of the degradation products from cytoplasmic and cell wall-associated β-galactosidase extracts from *B. longum* subsp. *longum* NCC 2705 (**a**) and *Bac. caccae* ATCC 43185 (**b**). Lane 1, galactose standard; lane 2 (β-1,3-galactobiose), 3 (β-1,4-galactobiose), 4 (β-1,6-galactobiose), substrate control without crude enzymes; lane 5, 6, 7, crude cytoplasmic enzyme incubated with β-1,3-galactobiose, β-1,4-galactobiose and β-1,6-galactobiose, respectively; lane 8, 9, 10, crude cell wall-associated enzyme incubated with β-1,3-galactobiose, β-1,4-galactobiose and β-1,6-galactobiose, respectively.

The TLC results show that when both strains were cultured in YC-AG, the cytoplasmic and cell wall-associated β-galactosidases from both strains were able to cleave β-1,3; 1,4; 1,6 linkages, whereas the β-galactosidase substrate specificity from *B. longum* subsp. *longum* NCC 2705 was weakest for the β-1,4 linkage, as the 1,4 linked substrate hydrolysis was incomplete (Figure 1).

**Figure 2:**
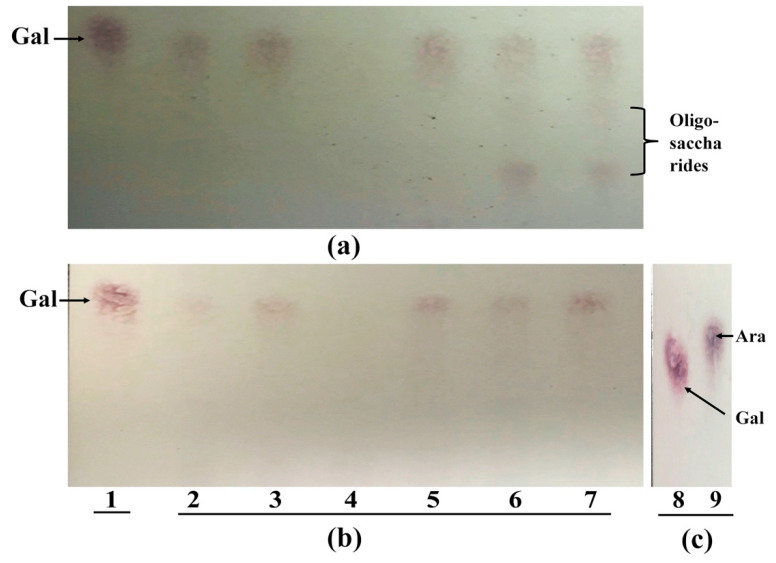
TLC analysis of the degradation products from cell wall-associated enzyme extracts from *B. longum* subsp. *longum* NCC 2705 (**a**) and *Bac. caccae* ATCC 43185 (**b**). Lane 1, galactose standard; lane 2, 3, 4, 5, 6, 7, crude cell wall-associated enzyme incubated with arabinogalactan (AG), de-arabinosylated AG (de-AG), gum arabic (GA), de-arabinosylated gum arabic (de-GA), galactan and de-arabinosylated potato galactan (de-galactan), respectively. Lane 8 and 9 from (**c**) means galactose and arabinose standard.

The cell wall-associated β-galactanases from both strains were able to release galactose from AG and de-arabinosylated AG (β-1,3;1,4;1,6 linkages), galactan and de-arabinosylated potato galactan (β-1,4 linkage), and de-arabinosylated GA (β-1,3;1,6 linkages) (Figure 2 and Figure S4). However, the enzyme extracts from both strains did not have any activity toward GA, which suggests that the arabinose-substituted side chains inhibited the enzymatic activity for β-1,3;1,6 linkages (Figure 2). These results indicate that both strains released galactose only from the β-1,4 linkage of AG. In addition, the enzymes (β-galactanase and/or β-galactosidase) from *Bac. caccae* ATCC 43185 released less galactose from AG and galactan compared to de-arabinosylated AG and de-arabinosylated potato galactan, suggesting that the enzyme activity for β-1,4 linkage was also inhibited by arabinose-substituted side chains (Figure 2b). As *B. longum* subsp. *longum* NCC 2705 had weak β-1,4 galactosidase activity, the released oligosaccharide likely has β-1,4 linkage (Figure 2a). However, there is no commercial standard available to confirm this.

**Figure 3:**
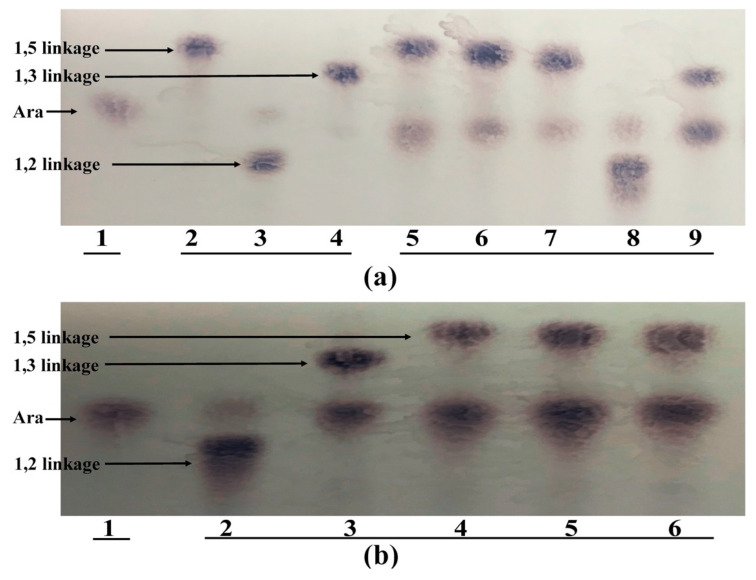
TLC analysis of the degradation products from cell wall-associated arabinosidase extracts from *B. longum* subsp. *longum* NCC 2705 (**a**) and *Bac. caccae* ATCC 43185 (**b**). For (**a**), lane 1: arabinose standard; lane 2: α-1,5-arabinobiose, 3: A2XX, 4: A3X, substrate control without crude enzymes; lane 5, 6, 7, 8, 9, crude cell wall-associated enzyme incubated with α-1,5 arabinobiose, α-1,5-arabinotriose, α-1,5-arabinotetraose, A2XX and A3X, respectively. For (**b**): lane 1, arabinose standard; lane 2, 3, 4, 5, 6, crude cell wall-associated enzyme incubated with A2XX, A3X, α-1,5-arabinobiose, α-1,5-arabinotriose and α-1,5-arabinotetraose.

To determine whether both strains had the ability to remove arabinose-substituted side chains from AG, cell wall-associated α-arabinosidase activities from both strains were tested. The arabinosidases from both strains were able to cleave α-1,2; 1,3 and 1,5 linkages (Figure 3). However, the enzyme activity from *B. longum* subsp. *longum* NCC 2705 was weaker for the α-1,5 linkage when the DP of α-1,5 substrates was higher (Figure 3a lanes 5–7). Moreover, the arabinosidase from both strains did not show any activity toward AG, GA, and galactan (Figure 2).

### 3.5. Growth in Mono- and Co-Culture on AG

The OD_600_ value of coculture of both strains (0.81 ± 0.08) was slightly higher than that of monoculture of *Bac. caccae* ATCC 43185 (0.60 ± 0.14) after incubating for 6 h, but slightly lower from 12 to 48 h (Figure S5). The pH of the cocultures was 5.75 ± 0.3, while the pH of each monoculture was higher at 6.15 ± 0.4 (*Bac. caccae* ATCC 43185) and 6.58 ± 0.2 (*B. longum* subsp. *longum* NCC 2705) after 12 h of incubation. After 48 h of fermentation, the pH of the cultures was slightly lower, at 5.71 ± 0.3 (coculture), 5.95 ± 0.3 (*Bac. caccae* ATCC 43185) and 6.08 ± 0.2 (*B. longum* subsp. *longum* NCC 2705). Between the 12- and 48-h incubation period, the OD_600_ value of the monoculture of *B. longum* subsp. *longum* NCC 2705 maintained the same level, whereas the pH of the monoculture decreased from 6.58 ± 0.20 to 6.08 ± 0.20.

**Figure 4:**
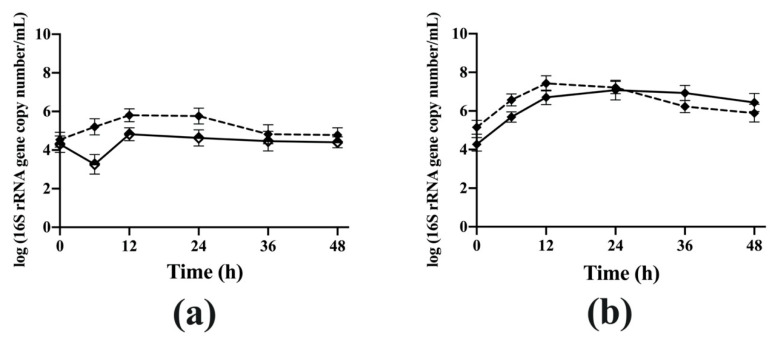
The quantification by ddPCR (**a**): 16S rRNA gene copy number of live *B. longum* subsp. *longum* NCC 2705; (**b**): 16S rRNA gene copy number of live *Bac. caccae* ATCC 43185) of monoculture *B. longum* subsp. *longum* NCC 2705 (half solid diamond) and *Bac. caccae* ATCC 43185 (diamond), and coculture of both strains (dashed line) in YC-AG medium. All experiments were done three independent times.

The ddPCR results showed that the 16S rRNA copy number of viable *B. longum* subsp. *longum* NCC 2705 in coculture was higher than that of monoculture during the whole incubation period (6 h: *p* < 0.05) (Figure 4a). However, there was no difference in the number of viable *Bac. caccae* ATCC 43185 between monoculture and coculture (Figure 4b). These results indicate that compared to monoculture, *B. longum* subsp. *longum* NCC 2705 showed higher abundance when it was cocultured with *Bac. caccae* ATCC 43185 in YC-AG. Furthermore, HPSEC results showed that 48% of AG (50 kDa-5 kDa) was degraded by the coculture after 48 h, which was higher than that of monoculture of either *B. longum* subsp. *longum* NCC 2705 (25%) or *Bac. caccae* ATCC 43185 (39%) (Figure S3).

**Figure 5:**
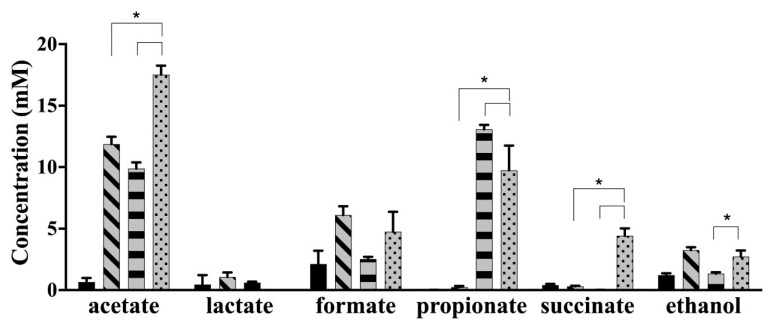
The concentration of short chain fatty acids (SCFAs) and other metabolites in monoculture of *B. longum* subsp. *longum* NCC 2705 (grey bar with diagonal lines), *Bac. caccae* ATCC 43185 (grey bar with horizontal lines), and coculture of both strains (grey bar with dots) in the YC-AG medium for 24 h (YC-AG before inoculation: black bar). Experiments were performed in triplicate. Dunnett’s multiple comparisons test was used to compare each monoculture to coculture (* indicates *p* < 0.05).

After 24 h of monoculture fermentation in YC-AG medium, both strains produced acetate (Figure 5). *B. longum* subsp. *longum* NCC 2705 and *Bac. caccae* ATCC 43185 produced 11.88 ± 0.61 mM and 9.89 ± 0.50 mM of acetate when they were monocultured in the YC-AG individually. However, 17.51 ± 0.75 mM of acetate was produced in the coculture, which is significantly higher (*p* < 0.05). When both strains were cocultured for 24 h in YC-AG medium, 4.41 ± 0.61 mM of succinate was produced, which was significantly higher than that produced after each monoculture (*B. longum* subsp. *longum* NCC 2705: 0.31 ± 0.05 mM; *Bac. caccae* ATCC 43185: 0.01 ± 0.01 mM) (*p* < 0.05). Furthermore, the concentration of ethanol in coculture (2.73 ± 0.49 mM) was significantly higher than that of monoculture of *Bac. caccae* ATCC 43185 (1.35 ± 0.12 mM) (*p* < 0.05). However, the coculture produced 9.75 ± 2.01 mM of propionate, which was significantly lower than that produced by *Bac. caccae* ATCC 43185 in the monoculture (13.07 ± 0.37 mM) (*p* < 0.05). Lactate was not observed in the coculture. There was no significant difference in formate production between mono- and co-culture of both strains over this time period (*p* > 0.05).

## 4. Discussion

AG must be degraded by extracellular enzymes to release mono- or oligo-saccharides for further transport and use [12,39]. β-galactanase is the main enzyme for degrading the backbone of AG. The genomes of both *B. longum* subsp. *longum* NCC 2705 and *Bac. caccae* ATCC 43185 encode β-galactanases. The β-galactanase of *B. longum* subsp. *longum* NCC 2705 is predicted to be exported outside the cytoplasmic membrane, due to the presence of a signal peptide and cell wall anchor motif [40]. The LPXTG membrane-anchoring motif has been found in some Gram-positive bacteria genomes that encode several surface proteins [8]. Although the β-galactanase gene from *Bac. caccae* ATCC 43185 does not have an LPXTG membrane-anchoring motif, it does have a signal peptide. Therefore, the β-galactanases from both *B. longum* subsp. *longum* NCC 2705 and *Bac. caccae* ATCC 43185 are predicted to be extracellular enzymes.

After the breakdown of the AG backbone, the oligosaccharides that are released from AG can then be either transported into the cell or hydrolyzed to monosaccharides by extracellular enzymes [8]. *Bacteroides* spp. are generally not efficient at transporting carbohydrate fragments into the cells after extracellular degradation [30]. However, the specific activity of β-galactosidase and α-arabinofuranosidase showed that both strains have extracellular oligosaccharide-degrading activities. In addition, analysis of substrate specificity showed that extracellular β-galactosidases and α-arabinofuranosidases of both strains are able to cleave all types of glycosidic linkages of AG. These results likely indicate that *Bac. caccae* ATCC 43185 has the ability and prefers external degradation of oligosaccharides that are released from AG. In contrast, the intracellular β-galactosidase activity of *B. longum* subsp. *longum* NCC 2705 is around 10 times higher than extracellular activity. Moreover, a three-gene cluster encoded in the genome of *B. longum* subsp. *longum* NCC 2705 is highly similar to the gene cluster that encodes an ABC-GOS uptake system in *B. breve* UCC 2003 [24]. These results imply that for GOS, *B. longum* subsp. *longum* NCC 2705 prefers internal degradation to sequester substrate and compete effectively with other gut bacteria.

β-galactosidase is only able to release galactose from the non-reducing end of the substrate, and may be inhibited by substituted side chains [41,42]. As the substrates used in this study have limited non-reducing ends, just a small amount of galactose could be released by β-galactosidase. If the endo-β-galactanase internally cleaves the main chain of AG to release more carbohydrate fragments with non-reducing ends, more galactose could be released from the non-reducing ends by β-galactosidase. However, the extracellular endo-β-galactanase activities for cleaving the galactans at β-1,3/1,6 linkages from both strains were inhibited by the arabinose side chains. Furthermore, the ability of the extracellular endo-β-galactanase of *Bac. caccae* ATCC 43185 to cleave the β-1,4 galactan was partially inhibited by the arabinosyl side chains. These findings suggest that without removing arabinose side chains from galactans, both strains are unable to degrade the β-1,3/1,6 linkages; only the β-1,4 linkages of the galactan can be cleaved. This means that type II AG is mostly unusable, and only part of type I AG can be cleaved at the β-1,4 linkage position by both strains without removing the arabinose side chains.

*Bac. caccae* ATCC 43185 had better growth on the de-arabinosylated GA compared to GA, which confirmed that the arabinose side chains had a negative effect on GA utilization by this strain. However, this growth stimulation was not observed for de-AG versus AG for *Bac. caccae* ATCC 43185. This likely resulted from the remaining arabinosyl side chains that were not completely removed from AG by using mild acid degradation. This method has only been validated when preparing the de-arabinosylated from of GA, but not for AG [35]. Furthermore, both de-AG and de-GA did not show any stimulation of the growth of *B. longum* subsp. *longum* NCC 2705. These results indicate that the DP of de-AG and de-GA is likely too long to be utilized effectively by *B. longum* subsp. *longum* NCC 2705. Therefore, AG utilization by *B. longum* subsp. *longum* NCC 2705 requires the contribution of other gut bacteria to provide lower DP oligosaccharides such as GOS.

As shown above, extracellular arabinosidase is very important to AG degradation. Both strains have extracellular α-arabinofuranosidase and β-arabinopyranosidase that are able to cleave α-1,2; 1,3; 1,5 linkages, when tested with low DP substrates. However, the extracellular arabinosidases from both strains were unable to release arabinose from AG, GA and galactan. These results indicate that the extracellular arabinosidases from both strains were not able to cleave arabinose from large MW substrates. One reason may be that large MW substrates have more complex structure, which could hide the non-reducing ends that are the preferred enzyme target sites.

The mono- and co-culture experiments showed that the cross-feeding of the partial breakdown products from AG occurred between *B. longum* subsp. *longum* NCC 2705 and *Bac. caccae* ATCC 43185 at the early fermentation stage (before 12 h of fermentation). The degraded AG fragments produced by extracellular enzymes from *Bac. caccae* ATCC 43185 may accumulate in the coculture and are likely to be utilized by *B. longum* subsp. *longum* NCC 2705 for growth. Growth of bifidobacteria can reduce the pH due to organic acid production, which results in restraining the growth of other less acid-tolerant bacteria [43]. Therefore, the pH of coculture was lower than that of *Bac. caccae* ATCC 43185 monoculture due to the metabolism of *B. longum* subsp. *longum* NCC 2705. *Bacteroides* species have poor growth at pH 5.5 due to lower acid tolerance [3]. The low pH condition inhibited the growth of *Bac. caccae* ATCC 43185, which may result in no further breakdown of carbohydrates in the coculture for *B. longum* subsp. *longum* NCC 2705 utilization. Therefore, the coculture of *B. longum* subsp. *longum* NCC 2705 and *Bac. caccae* ATCC 43185 showed a lower OD_600_ value than that of monoculture of *Bac. caccae* ATCC 43185. These results are consistent with one study that reported the abundance of *Bac. thetaiotaomicron* was maintained in the presence of bifidobacteria with high dilution rates during AG fermentation [30]. These results support the importance of pH in the gut environment for limiting the growth of *Bacteroides* species. In the distal colon where the pH would be optimal for the growth of *Bacteroides*, this species may release carbohydrate fragments of AG that can be utilized by other gut bacterial species, consequently influencing the composition of gut microbiota and their interactions. *Prevotella ruminicola* represents another fiber-degrading species that may participate in AG degradation in the gut, as this species has been enriched from cecal contents fermented with gum arabic [44], which has similar monosaccharide linkages to type II AG.

There is less information about the limitation of metabolic activity of *Bacteroides* species in low pH conditions. *Bacteroides* species ferment carbohydrates to mainly produce acetate, propionate and succinate [2]. However, the succinate can be converted to propionate via succinyl-, methylmalonyl-, and propionyl-coenzyme A by *Bac. fragilis* [45,46]. The concentration of succinate was significantly higher, whereas propionate was significantly lower in the coculture compared to *Bac. caccae* ATCC 43185 monoculture. These results imply that the metabolic activity of *Bac. caccae* ATCC 43185 was inhibited at low pH, which resulted in lower conversion of succinate to propionate. Therefore, the succinate accumulated in the coculture. The concentration of formate was higher than that of lactate in monoculture of *B. longum* subsp. *longum* NCC 2705, which correlates with nutrient limitation in the culture. Formate accumulates because extra ATP can be formed when bifidobacteria produce formate instead of lactate in a carbohydrate-limited environment [30]. The lactate that was mainly produced by *B. longum* subsp. *longum* NCC 2705 was not observed in the coculture, which was not surprising since lactate can be utilized by *Bacteroides* spp. [47,48]. The concentration of acetate was significantly higher in coculture compared to monoculture of *B. longum* subsp. *longum* NCC 2705 and *Bac. caccae* ATCC 43185. However, more evidence is required to demonstrate which strain contributed predominantly to the production of acetate.

## 5. Conclusions

In this study, we demonstrate why neither strain was able to completely degrade AG in pure culture even though annotated gene functions are present in the genome of both *B. longum* subsp. *longum* NCC 2705 and *Bac. caccae* ATCC 43185. This is because both strains are unable to remove arabinosyl side chains from AG, which resulted in inhibition of the endo-β-galactanase activity from both strains for cleavage of β-1,3;1,6 linkages of the AG backbone and side chains of type II AG. This suggests that both strains were unable to utilize type II AG without a complex consortium of strains with compatible enzymatic functions. The bifidogenic effect of AG was stimulated in the presence of *Bac. caccae* ATCC 43185, whereas the growth and metabolic activity of *Bac. caccae* ATCC 43185 were inhibited by the low pH of coculture due to the metabolism of *B. longum* subsp. *longum* NCC 2705. These results suggest that the cooperation between bifidobacteria and *Bacteroides* species may not occur in the proximal colon due to lower pH conditions than in the distal or descending colon. To better understand AG degradation by gut communities, the investigation of additional AG-degrading intestinal bacteria, such as *Prevotella* spp., is required. Both genomic and transcriptomic analysis of carbohydrate-degrading genes from these intestinal bacteria must be complemented with the study of enzyme activity.

## Figures and Tables

**Table 1:** Genus-specific primers used for quantification by droplet digital PCR (ddPCR).

Target Bacteria	Primers	Product Size	Tm ^1^	Target Gene	Reference
*Bac. caccae*	F: GTAACACGTATCCAACCTACC R: TATTCCTCACTGCTGCCTC	252	60 °C/30 s	16S rRNA	This study
*Bifidobacterium* spp.	F: TCGCGTCCGGTGTGAAAG R: CCACATCCAGCATCCAC	243	65 °C/25 s	16S rRNA	[37]

^1^ Tm: annealing temperature and time.

**Table 2:** Specific activity of the crude enzyme extracts from *B. longum* subsp. *longum* NCC 2705 and *Bac. caccae* ATCC 43185.

Substrate (*p*NP)	*B. longum* subsp. *longum* NCC 2705 Specific Activity (U/mg protein)		*Bac. caccae* ATCC 43185 Specific Activity (U/mg protein)	
	Cytoplasmic enzyme	Cell wall-associated enzyme	Cytoplasmic enzyme	Cell wall-associated enzyme
α-AF ^1^	0.15 ± 0.006 ^A,a^	0.19 ± 0.027 ^A,a^	0.01 ± 0.001 ^A,b^	0.09 ± 0.006 ^B,b^
β-AP ^2^	ND ^4^	0.05 ± 0.011 ^a^	ND	0.04 ± 0.004 ^a^
β-gal ^3^	2.64 ± 0.159 ^A,a^	0.22 ± 0.031 ^B,a^	0.21 ± 0.011 ^A,b^	0.11 ± 0.009 ^B,b^

^1^ α-AF: α-arabinofuranosidase, ^2^ β-AP: β-arabinopyranosidase. ^3^ β-gal: β-galactosidase. ^4^ ND: substrate cleavage was not detected. ^A,B^ Different upper-case letters mean statistical significance (*p* < 0.05) within each strain between the two cellular locations; ^a,b^ different lower-case letters indicate statistical significance of the Student’s *t*-test (*p* < 0.05) corresponding to the same cellular location in different strains (within rows). Enzyme assays were performed in triplicate.

## Supplementary Materials

The following are available online at https://www.mdpi.com/2076-2607/8/11/1703/s1, Table S1: Prediction of genes encoding AG degradation enzymes from the genomes of *B. longum* subsp. longum NCC 2705 and *Bac. caccae* ATCC 43185. Figure S1: Growth of *B. longum* subsp. *longum* NCC 2705 and *Bac. caccae* ATCC 43185 on YC medium with each carbon source: glucose, galactose, arabinose, AG, de-AG, GA, de-GA and without carbon source, respectively. Figure S2: HPSEC profile of standards. Figure S3. HPSEC profiles of carbohydrates in the residual YC-AG culture after 48 h of fermentation. Figure S4. Structure of AG, GA and potato galactan and specific target sites of substrate degrading enzymes. Figure S5. The growth curve of monoculture *B. longum* subsp. *longum* NCC 2705 and *Bac. caccae* ATCC 43185, and coculture of both strains in YC-AG medium.

## References

[1] Cockburn D.W., Koropatkin N.M. (2016). Polysaccharide degradation by the intestinal microbiota and its influence on human health and disease. J. Mol. Biol..

[2] Flint H.J., Duncan S.H., Scott K.P., Louis P. (2015). Links between diet, gut microbiota composition and gut metabolism. Proc. Nutr. Soc..

[3] Duncan S.H., Louis P., Thomson J.M., Flint H.J. (2009). The role of pH in determining the species composition of the human colonic microbiota. Environ. Microbiol..

[4] Kelly G.S. (1999). Larch arabinogalactan: Clinical relevance of a novel immune-enhancing polysaccharide. Altern. Med. Rev..

[5] Terpend K., Possemiers S., Daguet D., Marzorati M. (2013). Arabinogalactan and fructo-oligosaccharides have a different fermentation profile in the Simulator of the Human Intestinal Microbial Ecosystem (SHIME^®^). Environ. Microbiol. Rep..

[6] Yang L.-C., Lu T.-J., Lin W.-C. (2013). The prebiotic arabinogalactan of *Anoectochilus formosanus* prevents ovariectomy-induced osteoporosis in mice. J. Funct. Foods.

[7] Sakamoto T., Tanaka H., Nishimura Y., Ishimaru M., Kasai N. (2011). Characterization of an exo-β-1,3-d-galactanase from *Sphingomonas* sp. 24T and its application to structural analysis of larch wood arabinogalactan. Appl. Microbiol. Biotechnol..

[8] Fujita K., Sakaguchi T., Sakamoto A., Shimokawa M., Kitahara K. (2014). *Bifidobacterium longum* subsp. *longum* exo-β-1,3-galactanase, an enzyme for the degradation of type II arabinogalactan. Appl. Environ. Microbiol..

[9] Hinz S.W.A., Verhoef R., Schols H.A., Vincken J.-P., Voragen A.G.J. (2005). Type I arabinogalactan contains β-d-Galp-(1→3)-β-d-Galp structural elements. Carbohydr. Res..

[10] Cartmell A., Muñoz-Muñoz J., Briggs J.A., Ndeh D.A., Lowe E.C., Baslé A., Terrapon N., Stott K., Heunis T., Gray J. (2018). A surface endogalactanase in *Bacteroides thetaiotaomicron* confers keystone status for arabinogalactan degradation. Nat. Microbiol..

[11] Fujita K., Sasaki Y., Kitahara K. (2019). Degradation of plant arabinogalactan proteins by intestinal bacteria: Characteristics and functions of the enzymes involved. Appl. Microbiol. Biotechnol..

[12] Ndeh D., Gilbert H.J. (2018). Biochemistry of complex glycan depolymerisation by the human gut microbiota. FEMS Microbiol. Rev..

[13] Pokusaeva K., Fitzgerald G.F., van Sinderen D. (2011). Carbohydrate metabolism in bifidobacteria. Genes Nutr..

[14] Rivière A., Moens F., Selak M., Maes D., Weckx S., Vuyst L.D. (2014). The ability of bifidobacteria to degrade arabinoxylan oligosaccharide constituents and derived oligosaccharides is strain dependent. Appl. Environ. Microbiol..

[15] Hugenholtz F., Mullaney J.A., Kleerebezem M., Smidt H., Rosendale D.I. (2013). Modulation of the microbial fermentation in the gut by fermentable carbohydrates. Bioact. Carbohydr. Diet. Fibre.

[16] Flint H.J., Duncan S.H., Scott K.P., Louis P. (2007). Interactions and competition within the microbial community of the human colon: Links between diet and health. Environ. Microbiol..

[17] Hinz S.W.A., Pastink M.I., van den Broek L.A.M., Vincken J.-P., Voragen A.G.J. (2005). *Bifidobacterium longum* endogalactanase liberates galactotriose from type I galactans. Appl. Environ. Microbiol..

[18] Böger M., Hekelaar J., van Leeuwen S.S., Dijkhuizen L., Lammerts van Bueren A. (2019). Structural and functional characterization of a family GH53 β-1,4-galactanase from *Bacteroides thetaiotaomicron* that facilitates degradation of prebiotic galactooligosaccharides. J. Struct. Biol..

[19] Rivière A., Gagnon M., Weckx S., Roy D., Vuyst L.D. (2015). Mutual cross-feeding interactions between *Bifidobacterium longum* subsp. *longum* NCC2705 and *Eubacterium rectale* ATCC 33656 explain the bifidogenic and butyrogenic effects of arabinoxylan oligosaccharides. Appl. Environ. Microbiol..

[20] Shimokawa M., Kitahara K., Fujita K. (2015). Characterization of a β-L-arabinopyranosidase from *Bifidobacterium longum* subsp. longum. J. Appl. Glycosci..

[21] Bakir M.A., Kitahara M., Sakamoto M., Matsumoto M., Benno Y. (2006). *Bacteroides intestinalis* sp. nov., isolated from human faeces. Int. J. Syst. Evol. Microbiol..

[22] Kitahara M., Sakamoto M., Ike M., Sakata S., Benno Y. (2005). *Bacteroides plebeius* sp. nov. and *Bacteroides coprocola* sp. nov., isolated from human faeces. Int. J. Syst. Evol. Microbiol..

[23] Lan P.T.N., Sakamoto M., Sakata S., Benno Y. (2006). Bacteroides barnesiae sp. nov., *Bacteroides salanitronis* sp. nov. and *Bacteroides gallinarum* sp. nov., isolated from chicken caecum. Int. J. Syst. Evol. Microbiol..

[24] O’Connell Motherway M., Fitzgerald G.F., van Sinderen D. (2011). Metabolism of a plant derived galactose-containing polysaccharide by *Bifidobacterium breve* UCC2003. Microb. Biotechnol..

[25] Goulas T.K., Goulas A.K., Tzortzis G., Gibson G.R. (2007). Molecular cloning and comparative analysis of four β-galactosidase genes from *Bifidobacterium bifidum* NCIMB41171. Appl. Microbiol. Biotechnol..

[26] Møller P.L., Jørgensen F., Hansen O.C., Madsen S.M., Stougaard P. (2001). Intra- and extracellular β-galactosidases from *Bifidobacterium bifidum* and *B. infantis*: Molecular cloning, heterologous expression, and comparative characterization. Appl. Environ. Microbiol..

[27] Patel G.B., Mackenzie C.R., Agnew B.J. (1985). Properties and potential advantages of β-galactosidase from *Bacteroides polypraymatus*. Appl. Microbiol. Biotechnol..

[28] Scudder P., Uemura K., Dolby J., Fukuda M.N., Feizi T. (1983). Isolation and characterization of an endo-β-galactosidase from *Bacteroides fragilis*. Biochem. J..

[29] Tsai H.H., Hart C.A., Rhodes J.M. (1991). Production of mucin degrading sulphatase and glycosidases by *Bacteroides thetaiotaomicron*. Lett. Appl. Microbiol..

[30] Degnan B.A., Macfarlane G.T. (1995). Arabinogalactan utilization in continuous cultures of *Bifidobacterium longum*: Effect of co-culture with *Bacteroides thetaiotaomicron*. Anaerobe.

[31] Arboleya S., Watkins C., Stanton C., Ross R.P. (2016). Gut bifidobacteria populations in human health and aging. Front. Microbiol..

[32] Duncan S.H., Flint H.J. (2013). Probiotics and prebiotics and health in ageing populations. Maturitas.

[33] Rivière A., Selak M., Lantin D., Leroy F., Vuyst L.D. (2016). Bifidobacteria and butyrate-producing colon bacteria: Importance and strategies for their stimulation in the human gut. Front. Microbiol..

[34] Schell M.A., Karmirantzou M., Snel B., Vilanova D., Berger B., Pessi G., Zwahlen M.-C., Desiere F., Bork P., Delley M. (2002). The genome sequence of *Bifidobacterium longum* reflects its adaptation to the human gastrointestinal tract. Proc. Natl. Acad. Sci. USA.

[35] Ling N.X.-Y., Pettolino F., Liao M.-L., Bacic A. (2009). Preparation of a new chromogenic substrate to assay for β-galactanases that hydrolyse type II arabino-3,6-galactans. Carbohydr. Res..

[36] Wang Y., Kim J.Y., Park M.S., Ji G.E. (2012). Novel *Bifidobacterium* promoters selected through microarray analysis lead to constitutive high-level gene expression. J. Microbiol..

[37] Rinttilä T., Kassinen A., Malinen E., Krogius L., Palva A. (2004). Development of an extensive set of 16S rDNA-targeted primers for quantification of pathogenic and indigenous bacteria in faecal samples by real-time PCR. J. Appl. Microbiol..

[38] Polic I.I. (2018). Evaluation of the Impact of Azo Dyes on the Metabolism of Stabilized Fecal Communities and *In Vitro* Cell Culture. Master’s Thesis.

[39] Kaoutari A.E., Armougom F., Gordon J.I., Raoult D., Henrissat B. (2013). The abundance and variety of carbohydrate-active enzymes in the human gut microbiota. Nat. Rev. Microbiol..

[40] Von Heijne G. (1990). The signal peptide. J. Membr. Biol..

[41] Lammerts van Bueren A., Mulder M., van Leeuwen S., Dijkhuizen L. (2017). Prebiotic galactooligosaccharides activate mucin and pectic galactan utilization pathways in the human gut symbiont *Bacteroides thetaiotaomicron*. Sci. Rep..

[42] Sarbini S., Rastall R. (2011). Prebiotics: Metabolism, structure, and function. Func. Food Rev..

[43] Martinez F.A.C., Balciunas E.M., Converti A., Cotter P.D., de Souza Oliveira R.P. (2013). Bacteriocin production by *Bifidobacterium* spp. a review. Biotechnol. Adv..

[44] Kishimoto A., Ushida K., Phillips G.O., Ogasawara T., Sasaki Y. (2006). Identification of intestinal bacteria responsible for fermentation of gum arabic in pig model. Curr. Microbiol..

[45] Macy J.M., Ljungdahl L.G., Gottschalk G. (1978). Pathway of succinate and propionate formation in *Bacteroides fragilis*. J. Bacteriol..

[46] Rios-Covian D., Sánchez B., Salazar N., Martínez N., Redruello B., Gueimonde M., de los Reyes-Gavilán C.G. (2015). Different metabolic features of *Bacteroides fragilis* growing in the presence of glucose and exopolysaccharides of bifidobacteria. Front. Microbiol..

[47] Flint H.J., Scott K.P., Louis P., Duncan S.H. (2012). The role of the gut microbiota in nutrition and health. Nat. Rev. Gastroenterol. Hepatol..

[48] Schultz J.E., Breznak J.A. (1979). Cross-feeding of lactate between *Streptococcus lactis* and *Bacteroides* sp. isolated from termite hindguts. Appl. Environ. Microbiol..

