# Supplementary material for: Arabinogalactan Utilization by Bifidobacterium longum subsp. longum NCC 2705 and Bacteroides caccae ATCC 43185 in Monoculture and Coculture
Source: Microorganisms. 2020 Oct 31;8(11):1703. doi: 10.3390/microorganisms8111703 (PMC7693162; doi:10.3390/microorganisms8111703)
Supplement: Supplementary file 1 [file microorganisms-08-01703-s001.pdf]

Supplementary Material

Arabinogalactan Utilization by *Bifidobacterium longum* subsp. *longum* NCC 2705 and *Bacteroides caccae* ATCC 43185 in Monoculture and Coculture

Yan Wang [1] and Gisèle LaPointe [1,*]

[1] Canadian Research Institute for Food Safety, Department of Food Science, University of Guelph, Guelph, ON, N1G 2W1, Canada; ywang62@uoguelph.ca (Y.W.)

* Correspondence: glapoint@uoguelph.ca (G.L.); Tel.: +1-519-824-4120 Ext. 52099

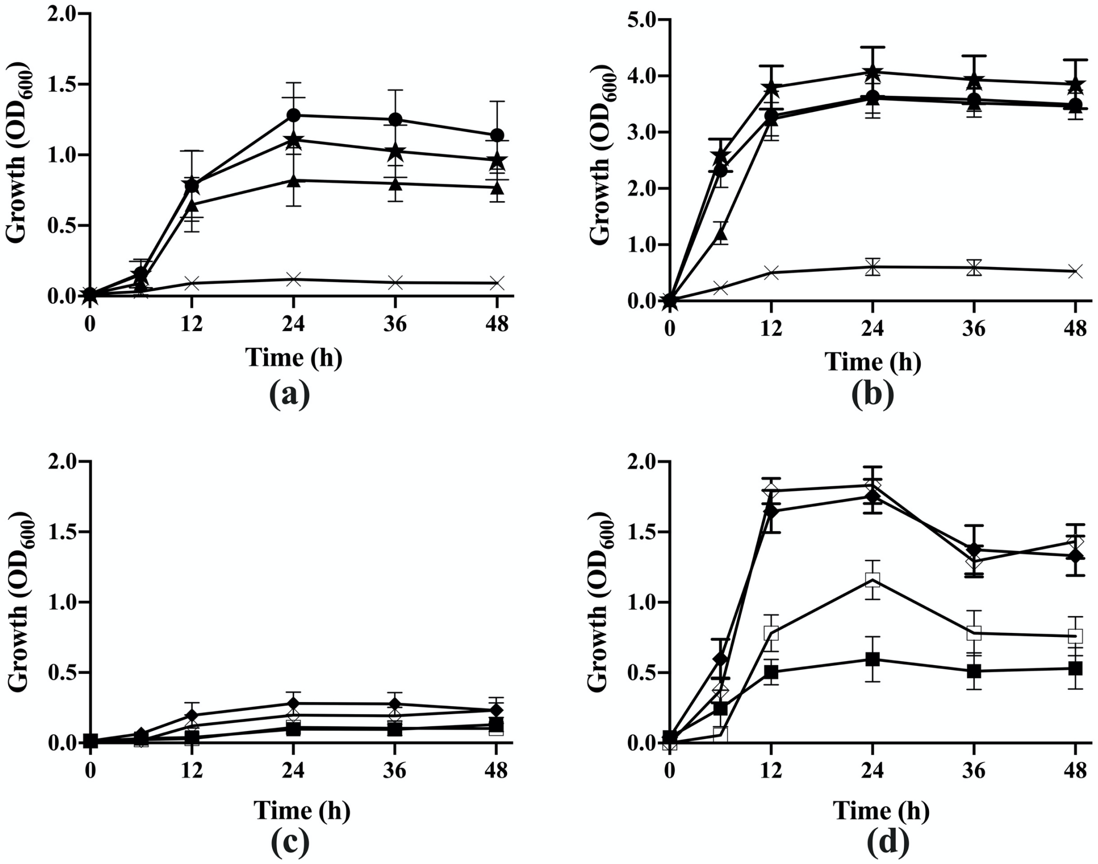

Figure S1. Growth of *B. longum* subsp. *longum* NCC 2705 (a; c) and *Bac. caccae* ATCC 43185 (b; d) on YC medium with each carbon source: glucose (black circle), galactose (black star), arabinose (black triangle), AG (black diamond), de-AG (hollow diamond), GA (black square), de-GA (hollow square) and without carbon source (×), respectively. All experiments were repeated independently three times.

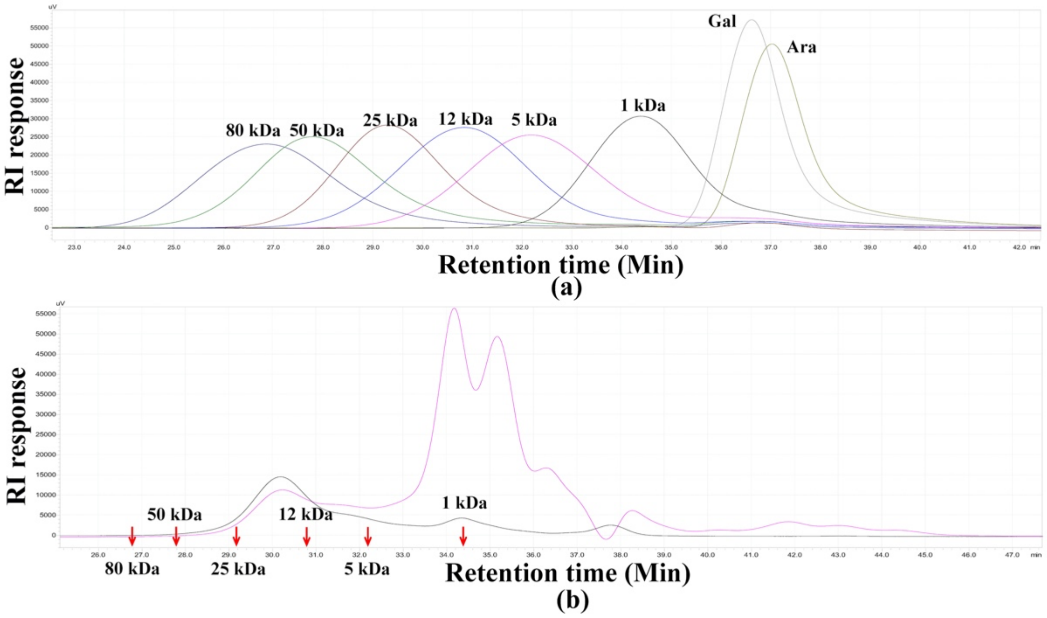

Figure S2. HPSEC profile of standards. a: dextran 80 kDa; dextran 50 kDa; dextran 25 kDa; dextran 12 kDa; dextran 5 kDa; dextran 1 kDa; Gal: galactose; Ara: arabinose. b: Black line: AG dissolved in water; pink line: AG dissolved in water (autoclaved).

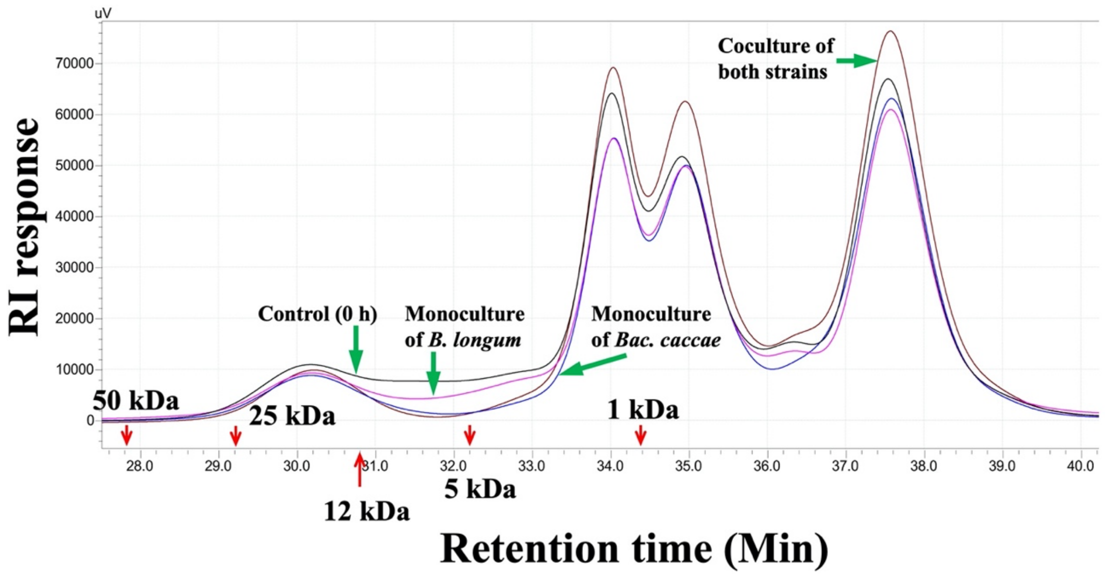

Figure S3. HPSEC profiles of carbohydrates in the residual YC-AG culture after 48 h of fermentation. Black line: 0 h; pink line: 48 h of fermentation of *B. longum* subsp. *longum* NCC 2705; blue line: 48 h of fermentation of *Bac. caccae* ATCC 43185; brown line, 48 h of fermentation of coculture *B. longum* subsp. *longum* NCC 2705 and *Bac. caccae* 43185. The retention time of standard peaks (Figure S4) are indicated by red arrows: dextran 50 kDa at 27.8 min; dextran 25 kDa at 29.2 min; dextran 12 kDa at 30.8 min; Dextran 5 kDa at 32.2 min; dextran 1 kDa at 34.4 min.

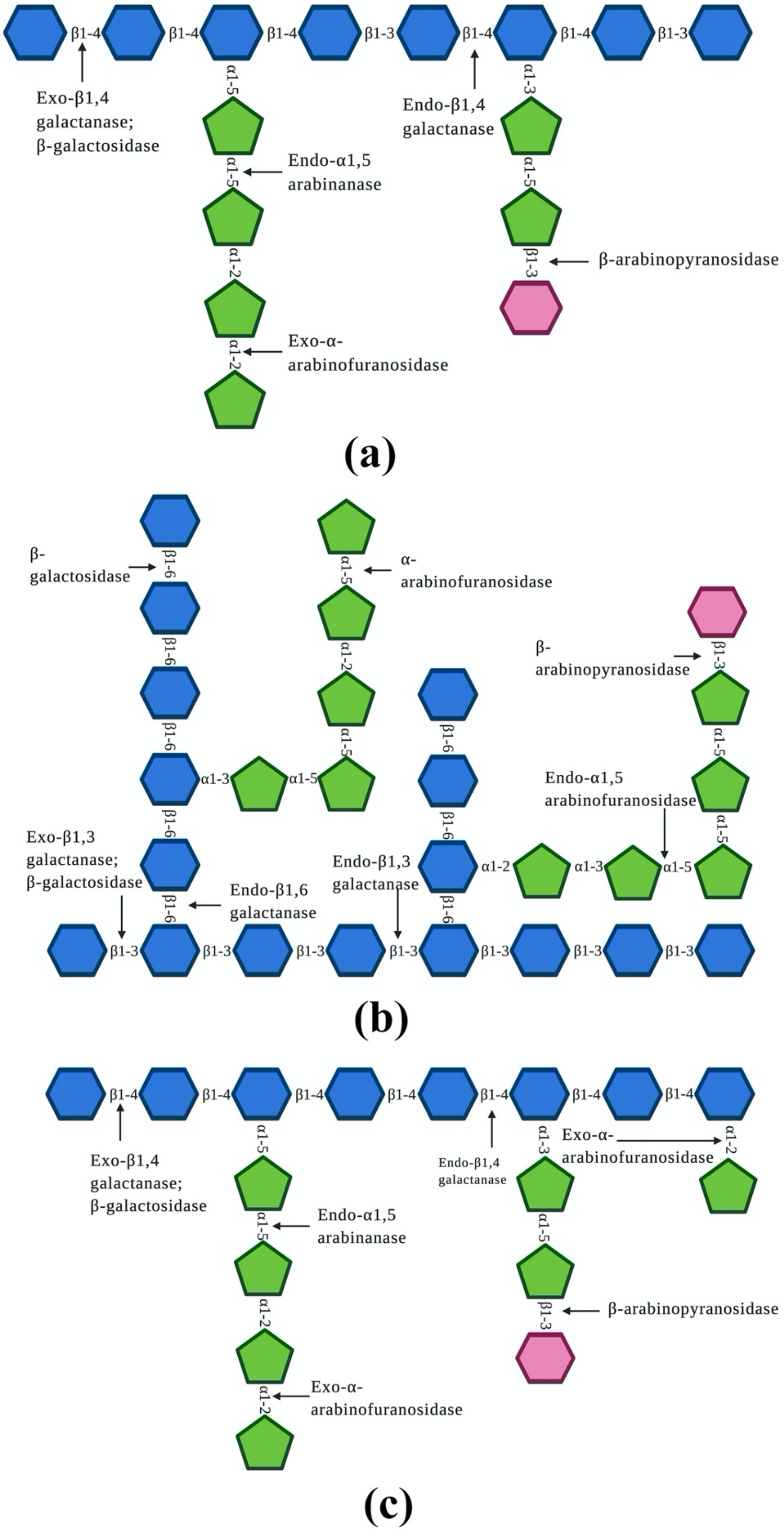

Figure S4. Structure of AG, GA and potato galactan and specific target sites of substrate degrading enzymes. a: type I AG; b: type II AG and GA; c: potato galactan. Blue hexagon: galactose; green pentagon: arabinofuranose; pink hexagon: arabinopyranose.

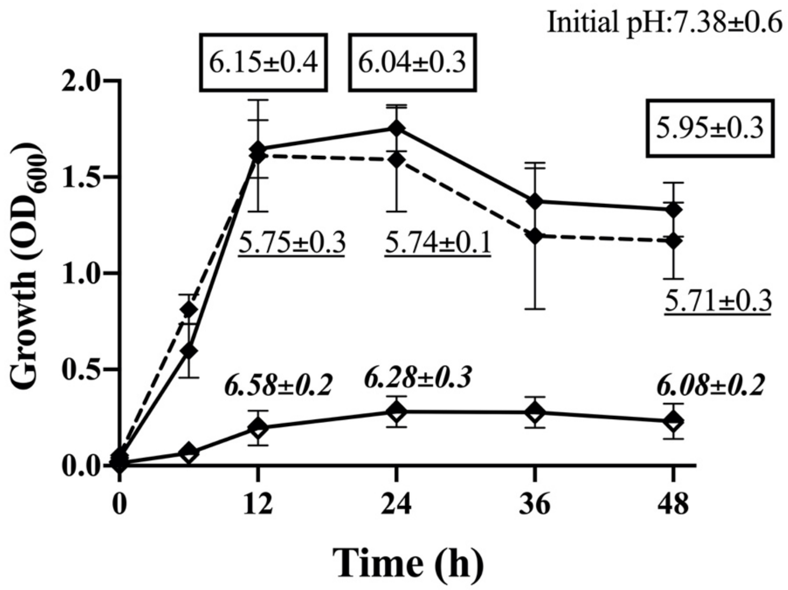

Figure S5. The growth curve of monoculture *B. longum* subsp. *longum* NCC 2705 (half solid diamond) and *Bac. caccae* ATCC 43185 (diamond), and coculture of both strains (dashed line) in YC-AG medium. The pH of monoculture of *B. longum* subsp. *longum* NCC 2705 is indicated by numbers within boxes and for *Bac. caccae* ATCC 43185 by underlined numbers; and coculture of both strains (numbers in bold and italic) at 12, 24 and 48 h fermentation in the YC-AG. All experiments were done three independent times.

Table S1. Prediction of genes encoding AG degradation enzymes from the genomes of *B. longum* subsp. *longum* NCC 2705 and *Bac. caccae* ATCC 43185.

Enzyme name	Enzyme ID	*B. longum* subsp. *longum* NCC 2705				*Bac. caccae* ATCC 43185			
		Locus Tag (BL)	Signal Peptide [1]	Motif/ Profile [2]	Predicted cellular location [3]	Locus Tag (BACCAC_)	Signal Peptide	Motif/ Profile	Predicted cellular location
β-galactanase	3.2.1.89	0257	Yes	LPXTG	Cell wall	02089	Yes	No	Unknown
β-galactosidase	3.2.1.23	0978	No	No	Cytoplasmic	00257	No	No	Cytoplasmic
		1168	No	No	Cytoplasmic	01243	No	No	Cytoplasmic
		0259	No	No	Unknown	01249	No	No	Unknown
		1775	No	No	Unknown	01322	No	No	Unknown
						01526	Yes	No	Unknown
						01713	No	No	Cytoplasmic membrane
						01783	No	No	Cytoplasmic
						01836	Yes	No	Unknown
						01971	No	No	Cytoplasmic
						02088	Yes	No	Unknown
						02615	No	No	Unknown
						02808	No	No	Cytoplasmic
						02846	Yes	No	Unknown
						02896	No	No	Unknown
						03226	Yes	No	Unknown
						03890	Yes	No	Unknown
α-arabinosidase	3.2.1.55	1166	No	No	Cytoplasmic	01784	No	No	Unknown
		0544	No	No	Cytoplasmic				
		1611	No	No	Cytoplasmic				
	3.2.1.99	0182	No	No	Unknown				
		0183	Yes	No	Unknown				

[1] Signal Peptide directs a protein into the secretory pathway. These secreted proteins can be classified into cytoplasmic or non-cytoplasmic proteins. [2] Motif & Profile indicates the presence of functional motifs that are used to infer protein specific localization. Non-cytoplasmic proteins can be further classified into cytoplasmic membrane, cell wall and extracellular protein in the Gram-positive bacteria; or cytoplasmic membrane, periplasmic, outer membrane and extracellular protein in Gram-negative bacteria. [3] Predicted cellular location is unknown means no site scored above 7.5.
